# A Case of Urogenital Human Schistosomiasis from a Non-endemic Area

**DOI:** 10.1371/journal.pntd.0004053

**Published:** 2015-11-05

**Authors:** Antonia Calvo-Cano, Lieselotte Cnops, Tine Huyse, Lisette van Lieshout, Josefina Pardos, M. E. Valls, Agustín Franco, David Rollinson, Joaquim Gascon

**Affiliations:** 1 ISGlobal, Barcelona Center of International Health Research (CRESIB), Hospital Clínic-Universitat de Barcelona, Barcelona, Spain; 2 Department of Clinical Sciences, Institute of Tropical Medicine, Antwerp, Belgium; 3 Department of Biology, Royal Museum for Central Africa, Tervuren, Belgium; 4 Department of Parasitology, Centre of Infectious Diseases, Leiden University Medical Centre, Leiden, The Netherlands; 5 Microbiology Department, Hospital Clínic, Barcelona, Spain; 6 Urology Department, Diagnostic Unit, Hospital Clínic, Barcelona, Spain; 7 Department of Life Sciences, Natural History Museum, London, United Kingdom; University of Queensland, AUSTRALIA

## Introduction

Schistosomiasis is a parasitic disease reported in 78 countries, with an additional recent outbreak in Corsica [1,2]. Generally, *Schistosoma haematobium* causes urogenital problems, whereas *S*. *mansoni*, *S*. *japonicum*, *S*. *mekongi*, *S*. *guineensis*, and *S*. *intercalatum* generate intestinal symptoms. Occasionally, ectopic tissue tropisms [3] and infections by parasites resulting from hybridization occur [4,5]. Geographical distribution and transmission of *Schistosoma* species depend on the presence of suitable intermediate snail hosts to complete the life cycle. Here we report on an unusual case of urogenital schistosomiasis in a Dominican adult male, living in Spain, with no history of visiting a known endemic area.

## Case Presentation

**Fig 1:**
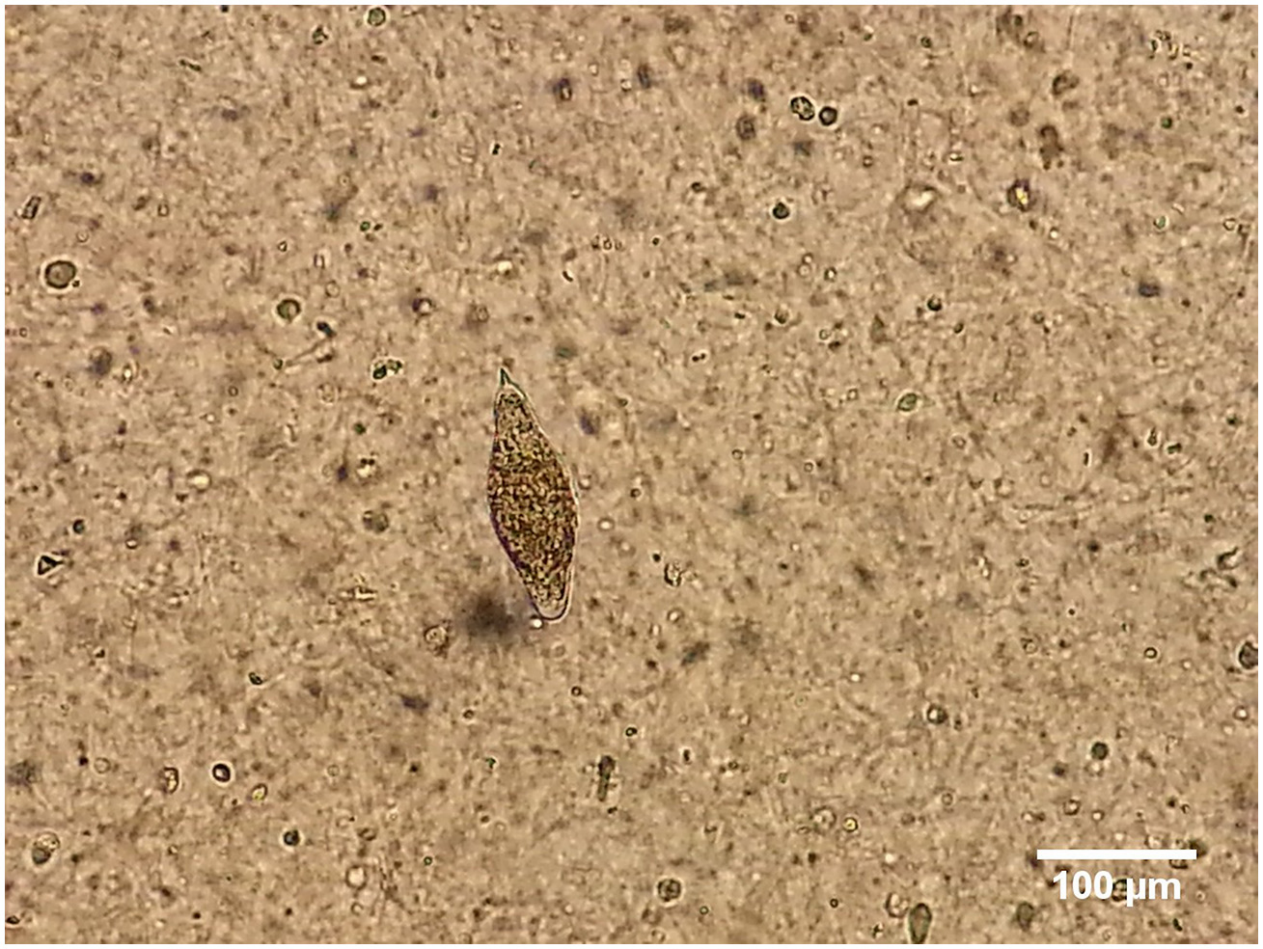
Morphology of the schistosome egg detected in a urine sample from the case report. Scale bar 100 μm.

In September 2013, a 45-year-old man presented at the outpatient clinic with intermittent dysuria, back pain, and haematuria. He reported a history of discontinuous haematuria since childhood and periodic dysuria and painful ejaculation in the last ten years. During this last decade, a urologic study was performed and indicated renal colic due to lithiasis. Born in the Dominican Republic, he often took baths in the Macasía River until 1984. One of his brothers died of renal failure, but he was unaware of cases of haematuria in the community. He had resided in Spain since September 2006. The only visits abroad were to Portugal in 2009 and to his homeland in 2010 and 2012. He had no history of sexually transmitted diseases, allergies, or chronic treatments. On examination, he had no urethral discharge or palpable inguinal adenopathies; abdomen and digital rectal examinations were normal. A urine sample, urethral smear, and blood sample were analyzed. A single dose of azithromycin (1 g, orally) was administered for empirical treatment of infectious urethritis without clinical improvement. Haematuria was confirmed (4–10 erythrocytes/μL), and tests for HIV, *Treponema pallidum*, *Mycobacteria* (urinary culture), *Chlamydia trachomatis* (PCR), and *Neisseria gonorrheae* (urethral smear) were negative. Blood count, coagulation, and biochemistry parameters were normal. A serologic test (Bilharziose Fumouze, indirect hemagglutination assays [IHA]) was positive (titer 1/80). Two out of three urine samples each showed a single viable schistosome egg of 164 and 155 μm long, respectively, with a terminal spine (Fig 1) typical of the *S*. *haematobium* group. Egg detection was negative in feces and semen. An abdominal ultrasound was normal, but cystoscopy revealed two exophytic lesions in the fundus of approximately 2 mm, whose biopsy did not show direct or indirect signs of the presence of *Schistosoma* eggs. Computed scanner (CT) urography excluded any other cause of haematuria. There was no evidence of calcification in the bladder. Real-time PCR was performed in two European reference laboratories on DNA extracts of two different urine collections. DNA was extracted with the MagNa Pure LC2.0 (Roche) using the Total Nucleic Acid Isolation High Performance Kit (Roche). The urine samples were positive with PCR targeting the *S*. *haematobium* complex-specific *Dra1* sequence [6] and the *Schistosoma* genus-specific fragment of the internal transcribed spacer (ITS) [7]. No signal was obtained in feces and serum with the *Dra1* PCR. The signal in urine was too weak to identify the causing species with an additional species-specific PCR [8], probably due to low DNA load. Feces, serum, and urine were negative with the *S*. *mansoni*-specific Sm1-7 real-time PCR [9]. Praziquantel was administered at 40 mg/kg/d for two days, with a complete recovery of urinary symptoms and no evidence of schistosome eggs in urine after 14 months of follow-up.

## Case Discussion

This report of urogenital schistosomiasis in an adult without bathing history in known endemic areas for *S*. *haematobium* is intriguing. Syndromic diagnosis is clear: positive serology, microscopic identification of terminal-spined eggs in two urine samples, and confirmation by two different PCR tests on two urine samples, together with the clinical picture and response to treatment. Etiological diagnosis is also clear: the causative agent of this case is not *S*. *mansoni* because of (1) the characteristic egg morphologies and (2) the positive PCR reaction for *S*. *haematobium* complex, and not for *S*. *mansoni*. Within the species of the *S*. *haematobium* complex, only *S*. *haematobium* or its hybrids with *S*. *bovis*, *S*. *intercalatum*, or *S*. *curassoni* can elicit urogenital disease [4,10]. The former is described in Africa, the Arabian Peninsula, and recently in Corsica [1], and hybrids have been reported in Cameroon and Senegal. Despite performing additional PCR tests, we were not able to confirm the species identification, although egg shape suggested the possibility of an *S*. *bovis*–*S*. *haematobium* hybrid [5].

Chronic symptoms since childhood make the Dominican Republic the most likely location for acquiring the infection. The patient was living until 1984 in Las Matas de Farfán, San Juan de la Maguana Province. The main economic activities of this village are agriculture and cattle farming. Although there is no record of *S*. *bovis* transmission nor *Bulinus* introduction in the area, some environmental factors might have facilitated autochthonous transmission of this species. From 1880, Lebanese migrants entered the Dominican Republic through the Haitian border, and have been established until today in San Juan de la Maguana. Lebanon was one of the countries with known transmission of *S*. *haematobium* [11]. In the 1970s and 1980s many dams were built in this province of the Dominican Republic, with irrigation of culture fields. This increase in freshwater habitats may have supported the introduction of a compatible snail host, giving rise to some limited transmission.

However, if schistosomiasis was acquired by the patient 30 years ago, there are several inconclusive factors: delay on diagnosis, the absence of other cases in the community except possibly his brother, discontinuation of symptoms, and lack of structural lesions associated with prolonged egg laying. There are old records of *Bulinus* in the south of Portugal and *Planorbarius metidjensis* in Spain that can transmit *S*. *bovis* [12]. The patient denied swimming in rivers or lakes in Portugal, but reported bathing in freshwater in Ciudad Real province in 2009. We interviewed three accompanying travelers who also bathed, but they have no symptoms.

## Conclusion

In summary, this is an unusual case report of schistosomiasis, both for its urinary tropism and its geographical origin. It is intriguing that other cases of urogenital schistosomiasis have not been recorded in the Dominican Republic nor in Spain. There is a need to establish whether there is a low prevalence of infection in associated communities and to screen freshwater habitats for compatible snail hosts.

## Ethics Statement

Written informed consent was given by the patient to publish clinical data.

**Key Learning Points**

- Despite many control efforts, schistosomiasis continues to be a threat to public health. Since the distribution of suitable vectors exceeds that of schistosomiasis, there are potential new transmission foci in many parts of the world.
- Clinicians should consider a diagnosis of urogenital schistosomiasis even when patients have not travelled to known endemic areas.
- Field surveys are needed to assess infection in associated communities and to screen freshwater habitats for compatible snail vectors.
